# Sociodemographic factors and early marriage among women in Bangladesh, Ghana and Iraq: An illustration from Multiple Indicator Cluster Survey

**DOI:** 10.1016/j.heliyon.2021.e07111

**Published:** 2021-05-25

**Authors:** Ahmed Abdus Saleh Saleheen, Sharmin Afrin, Samia Kabir, Md. Jakaria Habib, Maliha Afroj Zinnia, Md. Ismail Hossain, Iqramul Haq, Ashis Talukder

**Affiliations:** a Department of Statistics, Jagannath University, Dhaka, 1100, Bangladesh; b Department of Agribusiness and Marketing, Sher-e-Bangla Agricultural University, Dhaka, 1207, Bangladesh; c Department of Pharmacy, East West University, Dhaka, 1212, Bangladesh; d Department of Agricultural Statistics, Sher-e-Bangla Agricultural University, Dhaka, 1207, Bangladesh; e Statistics Discipline, Khulna University, Khulna, 9208, Bangladesh

**Keywords:** Early marriage, Low weight, Nutritional, Mortality, Unintended pregnancies

## Abstract

Early marriage is a form of violation of child rights to grow and develop. The Sustainable Development Goals had included early marriage in target 5.3, aiming to eliminate by 2030. This study examines the socio-demographic factors associated with women's early marriage in Bangladesh, Ghana, and Iraq using information extracted from 2019, 2017–2018, and 2018 Multiple Indicator Cluster Surveys (MICSs) of Bangladesh, Ghana, and Iraq, respectively. The chi-square test examined the association between socio-demographic factors and early marriage separately in all three countries. In logistic regression, key factors were primarily evaluated for determining effects on early marriage separately in all three countries. The mean age of the mother at first marriage was found to be 16.86, 20.23, and 20.05 years in Bangladesh, Ghana, and Iraq successively. According to surveys conducted in Bangladesh, Ghana, and Iraq, education levels of household heads and women, wealth status, mass media, number of household members, and residence were significant factors linked to early marriage. The odds of getting married early were significantly higher among women with no formal education and primary education than women with secondary or higher education in all three countries. In terms of economic status, a negative association was found between wealth status and early marriage in both Bangladesh and Ghana. Based on the findings, the study recommended that government take the necessary steps to reduce child marriage in all three countries by raising women's education and campaigning women by media to harmful effects of early marriage, particularly women from low-income families.

## 1 Introduction

Child marriage is a violation of human rights. It has an immense adverse effect on the children (especially girls) who enter into these marriages (Sah et al., 2014; Delprato et al., 2015; Kyari and Ayodele, 2014). Undoubtedly marriage is an essential ritual in the formation of a family which is socially and religiously recognized. But marriage should take place at a certain age when one is physically, mentally and morally self-sufficient. According to UNICEF, one should be married when he or she is 18 years of age or older; otherwise, this marriage is referred to as “Child Marriage” or “Early Marriage” (UNICEF, 2011; Walker, 2012).

Early marriage is a harmful practice in sexual health and well-being. Although the world is aware of the adverse effects of early marriage, the rate is still very high in lower- and middle-income countries (UNICEF, 2014). According to UNICEF, more than 12 million girls worldwide have been married at a young age (UNICEF, 2019). Of these 12 million, approximately 37% of early marriages occurred in sub-Saharan Africa and 30% in South Asia (UNICEF, 2019). Low educational attainment and poverty are prime reasons for this high rate of child marriage in lower- and middle-income countries (Montazeri et al., 2016). Though the prevalence of child marriage has declined in most developing countries, it has stagnated in Bangladesh (Sumon, 2014; Rahman, 2017), Ghana (de Groot et al., 2018), Iraq (Al-Ridhwany and Al-Jawadi, 2014) and many more countries.

Various studies of the Demographic Health Survey showed that marriage's average age is still below 18 years. Several socio-demographic and economic factors lead to marriage at such an early age (Kamal et al., 2014; Rumble et al., 2018). From the previous study, it was already known that lower educational and wealth status, religious bigotry and some more notable factors are there contributing to early marriage (Rumble et al., 2018; de Groot et al., 2018). It is a matter of sorrow that parents still believe that by marrying their daughters at an early age, they will be protected from sexual abuse and risk of sexually transmitted infections (STIs) (de Groot et al., 2018). But the reality presents an entirely different picture. The negative consequences of child marriage are awful. Negative health consequences of child marriage include poor maternal and reproductive health, increased risk of STIs, higher risk of intimate partner violence and more (Sarwade et al., 2019; Sanjeevi et al., 2018). This type of belief or misconception is only growing because of the lack of proper education.

In this study, we used three low- and middle-income countries: Bangladesh, Ghana, and Iraq. A question may arise as to why these three countries have been included in this study. Since child marriage still presents in developing countries. We need a clear idea about the current picture of early marriage in those countries. But every region and country have its view of marriage, and it varies from country to country, region to region. So, we have selected three low- and middle-income countries from three different regions where child marriage is more prevalent than others. It would be helpful to provide a comparison of these countries as well as low- and middle-income countries. Previous studies helped us to select the “more prevalent country” in this study (Sumon, 2014; Rahman, 2017; de Groot et al., 2018; Al-Ridhwany and Al-Jawadi, 2014).

From the above description, it is evident that child marriage is a major challenge with many different causes that need to be identified and explained, eliminating it's unfortunate and devasting effects. The Millennium Development Goals (preventing early, coerced, and forced marriage) first introduced the depletion of early marriage as a global priority in 2000, it still is a global agenda with the initiation of the Sustainable Development Goals (SDG) in 2015. Goal 5.3 specifically focuses on gender equality and empower women and girls, which monitors the progress level of eliminating early marriage (Svanemyr et al., 2015; United Nations, 2015). To achieve the goal of SDG, this study includes some objectives. The study's general objective was to find out the effects of socio-demographic factors associated with early marriage in three regions using the data from Bangladesh, Ghana, and Iraq 2019, 2017–2018, and 2018 multiple Indicators cluster surveys, respectively, and moving towards to SDGs target 5.3. The specific objectives were to examine the association between early marriage among women and its determinants and to assess the adjusted effects of selected covariates on early marriage among the three countries.

## 2 Materials and methods

### 2.1 Data source

The study used data from Multiple Indicator Cluster Survey (MICS) from three separate regions such as South Asia, West Africa, and the Middle East to determine early marriage (<18 years) and its associated factors. MICS was developed by the United Nations International Children's Fund (UNICEF) in the 1990s. It is a nationally representative cross-sectional household survey program designed to help countries collect data on various social and child health indicators.

For the South Asian region, we used data from the Bangladesh Multiple Indicator Cluster Survey (MICS), 2019 that has been conducted by the Bangladesh Bureau of Statistics (BBS) and UNICEF. Further, for West-African region data from Ghana Multiple Indicator Cluster Survey (MICS), 2017–18 were used that has been carried out by Ghana Statistical Service (GSS). And lastly, for Middle Eastern region data from Iraq Multiple Indicator Cluster Survey (MICS), 2018 were adapted that has been completed by the Central Statistical Organization (CSO) and Kurdistan Region Statistical Office (KRSO). The MICS uses a household questionnaire and women (age 15–49) questionnaire to assemble information about women and households with key indicators related to the Sustainable Development Goals (SDGs).

MICS used two-stage stratified cluster sampling in all their surveys. Since secondary data from three different regions have been used in this study, we are not discussing elaborately the data collection procedure. A more detailed description of the research environment, sampling methods and data collection procedures can be found on the MICS official website, which was “https://mics.unicef.org/”.

MICS used four types of questionnaires for data collection purpose, namely,

1. Household questionnaires
2. Woman's questionnaires
3. Man's questionnaires
4. Children's questionnaires

The questionnaire for women was administrated to women aged 15–49 years. The MICSs covered 64400, 13202, and 20521 residential households from Bangladesh, Ghana, and Iraq respectively. Consecutively 68709, 14609, and 31060 women aged 15–49 were qualified from these sampled households. The data weighted for this research purpose were provided with the MICS and the final sample size for this study is from several MICSs where 64,378 women aged 15–49 in Bangladesh, 14,374 women aged 15–49 in Ghana, 30,660 women aged 15–49 in Iraq.

### 2.2 Variables

The binary dependent variable of this study is the age of women respondents at first marriage which was documented in single years. The dependent variable was divided into two categories-those who were married before they reached 18 years of age were considered early married and the respondent who married at or after 18 years of age was considered lately married. According to the MICS report, eighteen years was selected as the cut-off point for defining the state of marriage as this is the legal female age at marriage (Bangladesh Bureau of Statistics (BBS) and UNICEF Bangladesh, 2019; Ghana Statistical Service, 2018). For the intent of the study, females who married before they reached age 18 were classified as “1” and “0” was used for the remainder.

Besides the dependent variable, we also considered a respondent's current age (15–24 years, 25–34 years, 35–49 years), residence (rural, urban), religion (Islam, others), wealth status (poor, middle, rich), sex of household head (male, female), women's education (no education, primary education, secondary and above education), Education of household head (no education, primary education, secondary and above education), mass media (exposed, not exposed), family size (1–3, 4–6, 7+) as potential factors for early marriage practice occurred in selected country.

### 2.3 Statistical analysis

Relevant statistical methods for analyzing the data were adopted to achieve different objectives of this study. Two-way contingency tables along with chi-square tests were employed to examine the relationships between socio-demographic factors and dependent variables and then examine the intercorrelation among independent variables. To get the adjusted effects of selected factors, we considered a statistical model appropriate for a binary response, namely the binary logistic regression model in multivariate setup (Lemeshow et al., 2013).

In a regression model, numerous statisticians and researchers have identified multicollinearity as high levels of interdependence among predictors, which in turn can be particularly difficult for studies (Thompson et al., 2017; Midi et al., 2010). At this point of tolerance, no formal cutoff value is there to dictate the presence of multicollinearity that can be used with tolerance (Midi et al., 2010). Based upon analysis, 0.1 or less tolerance value is considered as a cause of concern (Midi et al., 2010). Chiefly to dictate multicollinearity, a cutoff point of VIF 5 or 10 is suggested (Craney and Surles, 2002; Montgomery et al., 2012; Chatterjee and Hadi, 2013).

Let $Y_{i}$ denote the binary dependent variable for the $i^{th}$ observation,

(1) $$\text{here,}\;Y_{i}=\left\{ \begin{array}{l} 1,\;if\;marriage\;at\;<18\;age\;\\ 0,\;if\;marriage\;at\geq 18\;age \end{array}\right.$$

$X_{i1},\ldots ,X_{ip}$ be a set of explanatory variables which can be quantitative or indicator variables referring to the level of categorical variables.

Since Y is a binary variable, it has a Bernoulli distribution with parameter $\pi_{i}$. The dependent of the probability of success on independent variables is assumed to be respectively as-

(2) $$P\left(Y_{i}=1\right)=\pi_{i}=\frac{\text{exp}\left(\beta_{0}+\beta_{1}X_{i1}+\ldots +\beta_{p}X_{ip}\right)}{1+\text{exp}\left(\beta_{0}+\beta_{1}X_{i1}+\ldots +\beta_{p}X_{ip}\right)}$$

The above relation also can be expressed as,

(3) $$g\left(X\right)=logit\left(\pi_{i}\right)=log\frac{\pi_{i}}{1-\pi_{i}}=\beta_{0}+\beta_{1}X_{i1}+\ldots +\beta_{p}X_{ip}$$

The likelihood is maximized by finding estimates of the parameters that are most likely to give us the data. The maximum likelihood estimator (MLE) of $\beta_{0}$ and $\beta_{1}$ can be obtained by maximizing:

(4) $$L\left(\beta_{0},\beta_{1}\right)=\overset{n}{\underset{i=1}{\prod }}\frac{\text{exp}\left\{Y_{i}\left(\beta_{0}+\beta_{1}X_{i}\right)\right\}}{1+\text{exp}\left(\beta_{0}+\beta_{1}X_{i}\right)}$$

The result was presented as odds ratio (OR) and 95% confidence interval (CI).

For data management and analysis, the Statistical Package for Social Science (SPSS v25.0, IBM Corporation, Armonk, New York, NY, USA) software was used and R project for statistical computing version 4.0.0 was used for geographical mapping, using two most popular R-packages named “sf” (Pebesma, 2018) and “ggplot2” (Hadley, 2016).

### 2.4 Ethical statement

The study utilized data from Multiple Indicator Cluster Survey (MICS) from three separate regions that is publicly available in the following link: https://mics.unicef.org/. Therefore, this study does not need any additional ethical approval since the analysis was based on the freely available secondary data.

## 3 Results

### 3.1 Geographical conception of early marriage

**Figure 1:**
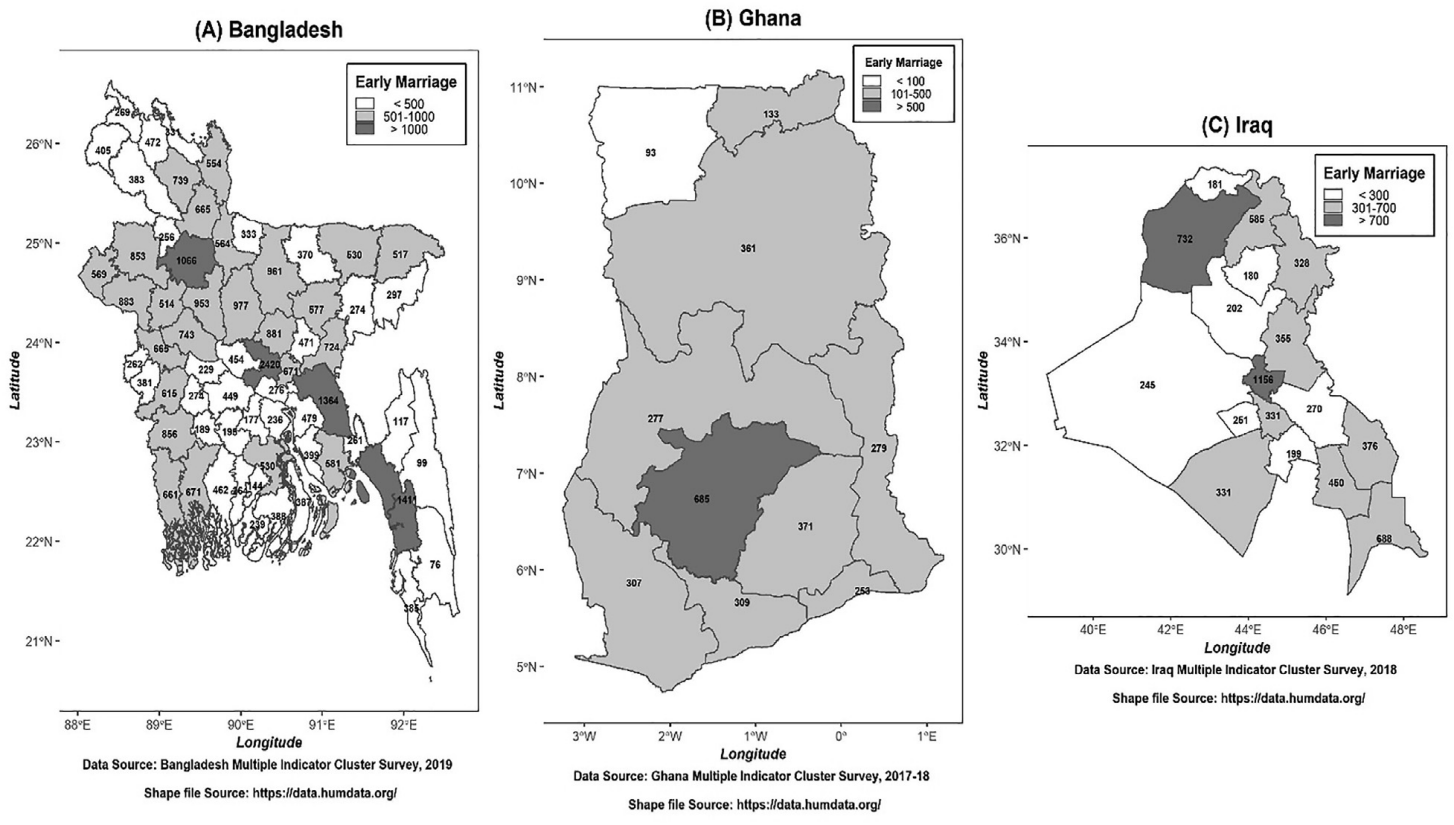
Geographical conception of early marriage in Bangladesh (A), Ghana (B), and Iraq (C). Shape file source for Bangladesh: https://data.humdata.org/dataset/administrative-boundaries-of-bangladesh-as-of-2015?fbclid=IwAR2lLqVrTlF9ig-Lq5NT0TqnA627KELdf2mjGn4JwV87-6bxxAbAL8JabpA. Shape file source for Ghana: https://data.humdata.org/dataset/ghana-administrative-boundaries?fbclid=IwAR3m3HAqS5i39S8r_ZVCguWqxhRHq3ZvGUOU1ZJQM9Q3YhIxc9F2SMRaYcw. Shape file source for Iraq: https://data.humdata.org/dataset/iraq-admin-level-1-boundaries?fbclid=IwAR0Recz_KnHYCy1TvCxJcJSt655v2cS1Tbg9d3wfXTmDThPVtbql7TRTXjo.

The geographical illustration of early marriage for all three distinct countries was expressed in Figure 1. Here, Bangladesh was divided into three distinct territories according to the occurrence of early marriage: red (>1000), orange (501–1000), and green (<500) (Figure 1A). Ghana was divided into three distinct territories as: red (>500), orange (101–500), and green (<100) (Figure 1B). And as for Iraq, the number of occurrences of early marriages varies according to red (>700), orange (301–700), and green (<300) (Figure 1C). Bangladesh was divided into districts, on the other hand, Ghana and Iraq were categorized into different regions.

The occurrence of early marriage in Bangladesh and the red zones consist of the districts with the largest number of early marriage occurrences (Figure 1A). As shown in (Figure 1A), most early marriages occurred in Dhaka, Chattogram, Cumilla, and Bogura. However (Figure 1B), illustrated the incidence of early marriage in the region of Ghana and Ashanti with the highest number of incidences and was identified as a red zone, which was much lower than in Bangladesh. And finally, early marriage experiences in Iraq, where the Capital Baghdad possessed the highest number of occurrences, which was also lower than that of the occurrence in Bangladesh (Figure 1C).

### 3.2 Mean age at first marriage for three country

The average age at first marriage for women found was 16.86 (SD 3.33), 20.23 (SD 5.05), and 20.05 (SD 4.84) in Bangladesh, Ghana, and Iraq successively.

### 3.3 Association between socio-demographic factors on early marriage

The prevalence of early marriage among women and different categories adopted is manifested in Table 1. In accordance with Table 1, the factors significantly associated (p < 0.001 or p < 0.05) with early marriage were women's current age, place of residence, religion, level of Education for both women and household head, wealth status, mass media exposure, family size for the survey of altogether three different countries (Bangladesh, Ghana, Iraq). At most Sex of household head was estimated to be significant (p < 0.001) in Bangladesh MICS data.

**Table 1:** Cross-tabulation analysis of early marriage among women by different background characteristics in Bangladesh, Ghana and Iraq.

Characteristics	Early Marriage (<18 years)					
	Bangladesh		Ghana		Iraq	
	No (%)	Yes (%)	No (%)	Yes (%)	No (%)	Yes (%)
**Current Age**						
15–24	30.2	69.8	46.3	53.7	38.2	61.8
25–34	39.3	60.7	71.2	28.8	69.2	30.8
35–49	34.5	65.5	70.9	29.1	76.2	23.8
**Effect Size (p-value)**	0.073 (p < 0.001)		0.176 (p < 0.001)		0.304 (p < 0.001)	

**Residence**						
Urban	40.9	59.1	74.1	25.9	67.4	32.6
Rural	33.5	66.5	62.3	37.7	62.5	37.5
**Effect Size (p-value)**	0.064 (p < 0.001)		0.127 (p < 0.001)		0.047 (p < 0.001)	

**Religion**						
Islam	34.2	65.8	65.1	34.9	65.9	34.1
Others	44.9	55.1	68.7	31.3	76.9	23.1
**Effect Size (p-value)**	0.066 (p < 0.001)		0.031 (p < 0.001)		0.015 (p < 0.05)	

**Wealth status**						
Poor	30.5	69.5	58.3	41.7	61.3	38.7
Middle	31.6	68.4	63.6	36.4	65.0	35.0
Rich	41.2	58.8	78.3	21.7	70.9	29.1
**Effect Size (p-value)**	0.107 (p < 0.001)		0.197 (p < 0.001)		0.091 (p < 0.001)	

**Sex (HH)**						
Male	34.8	65.2	67.5	32.5	66.1	33.9
Female	38.9	61.1	69.0	31.0	64.1	35.9
**Effect Size (p-value)**	0.027 (p < 0.001)		0.015 (p = 0.07)		0.011 (p = 0.06)	

**Women's education**						
No education	28.9	71.1	58.6	41.4	59.9	40.1
Primary	28.0	72.0	66.6	33.4	59.8	40.2
Secondary+	40.6	59.4	88.7	11.3	75.1	24.9
**Effect Size (p-value)**	0.128 (p < 0.001)		0.205 (p < 0.001)		0.158 (p < 0.001)	

**Education (HH)**						
No education	31.0	69.0	59.0	41.0	61.6	38.4
Primary	31.0	69.0	67.1	32.9	61.9	38.1
Secondary+	41.6	58.4	81.5	18.5	70.1	29.9
**Effect Size (p-value)**	0.109 (p < 0.001)		0.162 (p < 0.001)		0.087 (p < 0.001)	

**Mass media**						
Exposed	65.7	34.3	90.0	10.0	80.4	19.6
Not exposed	33.2	66.8	60.9	39.1	62.1	37.9
**Effect Size (p-value)**	0.058 (p < 0.001)		0.182 (p < 0.001)		0.111 (p < 0.001)	

**Family size**						
1–3	34.8	65.2	74.7	25.3	72.9	27.1
4–6	34.1	65.9	69.8	30.2	71.0	29.0
≥7	40.7	59.3	61.2	38.8	61.2	38.8
**Effect Size (p-value)**	0.048 (p < 0.001)		0.108 (p < 0.001)		0.107 (p < 0.001)	

HH = Household Head.

### 3.4 Collinearity diagnostic and identify the contributing factors

It should be noted that multicollinearity is one of the assumptions for implementing any regression model. The presence of multicollinearity might reduce the precision of estimated coefficients and also, the estimation of coefficients depends on other independent variables in the model. For this reason, we checked multicollinearity before performing the regression models. we observed that there was no multicollinearity present in this analysis. All the variables of the current analysis possessed tolerance values above 0.1 and VIF values less than 2.5.

Table 2 also demonstrates the risk factors associated with child marriage, estimated using three separate binary logistic regression models. There was a significant negative association between women age and early marriage. The result indicated that Iraq (OR = 4.54, 95% CI = 3.41–6.05) had significantly highest odd of early marriage among women aged 15–19 years followed by Ghana (OR = 3.01, 95% CI = 2.30–3.93) and Bangladesh (OR = 1.47, 95% CI = 1.36–1.63) compared to women aged 35–49 years. In Bangladesh, women aged 15–24 years were 47 percent (OR = 1.47, 95% CI = 1.36–1.63) more likely to marry before 18 years of age than women aged 35–49 years. By Ghana MICS 2017–18, the incidence of early marriage in 25–34 years women was accounted to be 1.27 times (OR = 1.27, 95% CI = 1.05–1.54) more than women aged 35–49 years.

**Table 2:** Binary logistic regression analysis showing the effect of early marriage among women by background characteristics in Bangladesh, Ghana and Iraq.

Characteristics	Early Marriage (<18 years)					
	Bangladesh		Ghana		Iraq	
	OR	95% CI	OR	95% CI	OR	95% CI
**Current Age**						
15–24	1.47∗∗∗	1.36–1.63	3.01∗∗∗	2.30–3.93	4.54∗∗∗	3.41–6.05
25–34	.94	0.88–1.01	1.27∗	1.05–1.54	1.23	0.97–1.56
35-49 (ref.)	1		1		1	
**Residence**						
Urban	1.00	0.90–1.10	0.86	0.69–1.06	0.71∗∗	0.56–0.89
Rural (ref.)	1		1		1	
**Religion**						
Islam	1.75∗∗∗	1.58–1.95	1.04	0.84–1.28	1.03	0.35–3.07
Others (ref.)	1		1		1	
**Wealth status**						
Poor	1.18∗∗∗	1.08–1.29	1.49∗	1.09–2.02	0.80	0.62–1.05
Middle	1.19∗∗∗	1.07–1.31	1.48∗	1.06–2.07	1.29	0.96–1.73
Rich (ref.)	1		1		1	
**Sex (HH)**						
Male	1.17∗∗	1.06–1.29	0.98	0.80–1.19	1.39	0.91–2.13
Female (ref.)	1		1		1	
**Women's education**						
No education	1.49∗∗∗	1.36–1.64	2.69∗∗∗	1.64–4.40	1.97∗∗∗	1.42–2.72
Primary	1.50∗∗∗	1.39–1.63	2.16∗∗∗	1.36–3.43	1.49∗∗	1.14–1.96
Secondary+ (ref.)	1		1		1	
**Education (HH)**						
No education	0.96	0.88–1.04	1.01	0.70–1.17	0.71∗	0.51–0.98
Primary	1.06	0.98–1.15	1.04	0.74–1.47	1.27∗	1.00–1.63
Secondary+ (ref.)	1		1		1	
**Mass media**						
Exposed	0.35∗∗∗	0.25–0.51	0.52∗	0.31–0.87	0.58∗	0.38–0.89
Not exposed (ref.)	1		1		1	
**Family size**						
1–3	1.27∗∗∗	1.15–1.41	0.61∗∗∗	0.46–0.80	0.51∗	0.30–0.86
4–6	1.31∗∗∗	1.20–1.42	0.82∗∗	0.68–0.99	0.63∗∗∗	0.50–0.79
≥7 (ref.)	1		1		1	
**Pseudo R-square**	0.023		0.083		0.115	

HH = Household Head; (ref.) = Reference Category; Statistical Significance: ∗p < 0.05, ∗∗p < 0.01, ∗∗∗p < 0.001.

Urban women in Iraq were less likely to marry at a very young age as compared with rural women (OR = 0.71, 95% CI: 0.56–0.89). Additionally, the findings from this study revealed that Muslim women in Bangladesh were 1.75 times (OR = 1.75, 95% CI = 1.58–1.95) more likely to be involved in early marriage compared to non-Muslim women.

Among the wealth status, findings also reveal that the rate of engaging in early marriage increases with a decrease in wealth. In Bangladesh, poor and middle-class families had 18 percent (OR = 1.18, 95% CI = 1.08–1.29) and 19 percent (OR = 1.19, 95% CI = 1.07–1.31) more likely to be involved in early marriage than wealthy families respectively. Even in Ghana, women from poor and middle households were more tendency to get married early than women from wealthy households. For Ghana, the odds indicate that women from poor and middle households were 49 percent (OR = 1.49, 95% CI = 1.09–2.02) and 48 percent (OR = 1.48, 95% CI = 1.06–2.07) more likely to get married before 18 years of age than women from rich households. Sex of household head was significantly associated with early marriage only for Bangladesh. Male household heads were 17 percent (OR = 1.17, 95% CI = 1.06–1.29) more likely to get married before 18 years of age compared with the female household heads.

In all three countries, the logistic regression analysis revealed that there was a significant negative association between early marriage and women education. The result from these three countries all confirmed that women with no education or primary education were more likely to engage in early marriage than women with secondary or higher education. Among the three countries Ghana (OR = 2.69, 95% CI = 1.64–4.40) had significantly highest odd of early marriage among women with no formal education followed by Iraq (OR = 1.97, 95% CI = 1.42–2.72) and Bangladesh (OR = 1.49, 95% CI = 1.36–1.64) compared to women with secondary and above educational status as a reference category. In terms of primary education, the result indicates that Ghana (OR = 2.16, 95% CI = 1.36–3.43) had significantly highest odds of early marriage among women with attained primary education followed by Bangladesh (OR = 1.50, 95% CI = 1.39–1.63) and Iraq (OR = 1.49, 95% CI = 1.14–1.96) compared to women with secondary and above educational status as a reference category.

In Iraq, the household head had no education (OR = 0.71, 95% CI = 0.51–0.98) were lower odds of early marriage as compared with a household head who had secondary and above education, while primary educated household head were 1.27 times (OR = 1.27, 95% CI = 1.00–1.63) more likely to be involved in early marriage compared to household head with secondary and above education.

With regards to mass media in all three countries, it has been observed that women who were exposed to media were less likely to be involved in early marriage than women who have not exposed to any media at all. Moreover, for the two countries (Ghana and Iraq), It was also interesting to find that early marriage less occurred in a family with fewer members. Also, nuclear family size (1–3) was 39 percent and 49 percent less likely to be involved in early marriage than extended families (≥7) in Ghana and Iraq respectively. In contrast to Bangladesh, the results indicate that nuclear families were more likely to get married before 18 years of age (OR = 1.27, 95% CI = 1.51–1.41) than compared to the extended families (≥7).

## 4 Discussion

This study was designed to identify the fundamental factors contributing to early marriage in three different countries through the data extracted from the MICSs of Bangladesh, Ghana, and Iraq in 2019, 2017–2018, and 2018, respectively. This section summarizes the findings and contributions made.

The sample sizes we adopted in this study for Bangladesh were 64378, for Ghana 14374, and Iraq 30660. The ratio of early marriage in terms of sample size are 54.1 percent, 21.3 percent, and 23.2 percent respectively. Among the three countries we studied, Bangladesh is interestingly found to have the largest number of early marriage phenomenon. One possible reason for this may be the sample size number that we used in this study.

The current study found that mean age at the first union in Ghana and Iraq is higher than in Bangladesh. It is evident from the analysis that early marriage for all three countries decreases with age, thereby suggesting that older women were more reluctant to marry at a very young age than their younger counterparts. A similar conclusion was reached by another researchers. They have demonstrated the interrelation between age and marriage at a younger age (Kumchulesi et al., 2011; Palamuleni, 2011; Adebowale et al., 2012).

Place of residence is a crucial factor in the pattern of the Early Marriage (Hotchkiss et al., 2016). But this study could not detect significant influence on early marriage in Bangladesh and Ghana. In Iraq, urban women were significantly less likely to practice early marriage than rural women. Some former studies exhibited the conservativeness of the rural residential area as well (Rahman, 2017; Rumble et al., 2018).

Supporting our study, education of both women and household head were a vital factor for governing early marriage in Bangladesh, Ghana, and Iraq sequentially. Our findings confirmed that women without any education or who attained primary education had more tendency to experience early marriage than higher educated women. This particular result is in line with the majority of South Asian literature and elsewhere (Bezie and Addisu, 2019; Wodon et al., 2015). In Bangladesh, women holding higher education were less likely to get married early than uneducated women (Biswas et al., 2019). Because higher education contributes to better information on marital age and marital health and thus helps to empower women (Porter, 2013; Duflo, 2012; Mim, 2017). Also, highly educated women are more likely to participate in technical and other job-related activities, and thus want to postpone marriage. Bezie and Addisu (2019) and Talukder et al. (2020) have also found an affirmative relationship between female education and age at the first union in Bangladesh and elsewhere.

Our research was unable to discern a major impact in terms of the educational status of heads of households except for Iraq. An interesting finding showed that uneducated household heads were more careful about the age at first marriage in Iraq. However primary educated heads of households were more conservative than highly educated heads of households (Bezie and Addisu, 2019).

Muslim families showed greater chances of early marriage compared to non-Muslim families and these findings are directly in line with previous findings (Rahman, 2017; Kamal et al., 2014). This superior outcome of our study indicated that, despite decades, Muslims' attitude toward the age of first marriage has not changed. Even massive Muslim families, especially in rural areas, believe that early marriage is right, although many Muslim countries have changed this pattern (Maaike, 2016). Other studies conducted in Bangladesh also suggested that Muslim households are likely to have more approval rate for early marriage (Islam et al., 2017; Islam and Gagnon, 2014).

Our analysis revealed a significant relation between early marriage and media-exposed women. Mass media access (radio/TV/newspaper) has a significant impact on marriage age and therefore, can be used to raise awareness of this issue (Rumble et al., 2018; Jain et al., 2011). Concerning the exposure to deliver messages through various forms of mass media, the results were found to be significant for all three countries. Of the respondents, those who were not exposed to the mass media were more likely to marry before the age of 18 than those who were exposed to the media, and this result is consistent with the earlier study conducted in Bangladesh (Sultana et al., 2015).

The major factor observed in all three survey regions were family size. It was identified from the study child marriage decreases at a nuclear family compared to extended family (>6 members). However, when comparing our results to older study in Ethiopia, it must be pointed out that the association between family size and marital age was significant and occurrence of early marriage were higher among family with large member (Bezie and Addisu, 2019).

Concerning the family wealth status, it has been noted that women from poor and middle households are more prone to be married at less than 18 years of age than women of rich quintile in Bangladesh and Ghana. Analogous findings were found in studies that have been carried out in Iran, Bangladesh and East Africa (Montazeri et al., 2016; Kamal, 2012; Schaffnit et al., 2019). Early marriage is less common in countries with well employment opportunities for women (Otoo-Oyortey and Pobi, 2003). Though no significant effect of wealth quintiles was observed in Iraq and this was not in line with another study (Stark, 2017).

In the case of the sex of household heads, results from our study showed child marriage was common in patriarchal families in comparison with the matriarchal family in Bangladesh. Yet, no significant impact of household head gender was observed in the other two countries.

Although this study leads to good results, it suffers from some limitations. One limitation of our study is that we did not consider equal sample size. However Multiple Indicator Cluster Survey data is not available for all country and too difficult to find the equal sample size during MICS6 survey. Another limitation of our study is that our data was cross-sectional data and then it is difficult to identify a causal relationship.

## 5 Conclusions

In this study we compare three Low-and Middle-income countries (LMIC), Bangladesh, Iraq, and Ghana from South Asia, the transcontinental region, and West Africa, respectively, to assess the actual condition of early marriage in the selected LMIC's. Among the three countries, Bangladesh had a lower median age at first marriage. In summary, we conclude that a respondent's current age, wealth status, women education, mass media, and family size are the significant determinates of early marriage in all three countries. However, sex of household head and religion were found to be significant factors only in Bangladesh. Rich-family women were more likely to involve late marriage nearly in Bangladesh and Ghana while the residence is an only significant factor in reducing early marriage in Iraq. Awareness rising program plays a vital role in the decline of early marriage. Policymakers should take the necessary steps to reduce early marriage through mass media for the expansion of women's education especially women were from a poor household in both Bangladesh and Ghana. The results of the study strongly recommend efforts to reduce early marriage by enhancing women's empowerment in Bangladesh. However Early marriage is a critical problem among women in all three countries. At the moment, early marriage is one of the vital issues in under-developed and developing countries and should be eradicated within 2030 which is the SDGs target 5.3. So, it will be a wise decision to move forward with significant factors to acquire sustainable development goals.

## Declarations

### Author contribution statement

Iqramul Haq: Conceived and designed the experiments; Performed the experiments; Contributed reagents, materials, analysis tools or data.

Ahmed Abdus Saleh Saleheen, Samia Kabir, Md. Jakaria Habib: Performed the experiments; Analyzed and interpreted the data.

Sharmin Afrin: Contributed reagents, materials, analysis tools or data; Wrote the paper.

Maliha Afroj Zinnia: Performed the experiments; Wrote the paper.

Md. Ismail Hossain: Performed the experiments; Analyzed and interpreted the data; Wrote the paper.

Ashis Talukder: Contributed reagents, materials, analysis tools or data; Wrote the paper.

### Funding statement

This research did not receive any specific grant from funding agencies in the public, commercial, or not-for-profit sectors.

### Data availability statement

Data associated with this study is available at: https://mics.unicef.org/.

### Declaration of interests statement

The authors declare no conflict of interest.

### Additional information

No additional information is available for this paper.
